# Parosteal Osteoma of the Clavicle

**DOI:** 10.1155/2014/824959

**Published:** 2014-08-25

**Authors:** Takao Inokuchi, Toshiaki Hitora, Yosiki Yamagami, Hideki Nishimura, Tetsuji Yamamoto

**Affiliations:** Department of Orthopaedic Surgery, Kagawa University, 1750-1 Ikenobe, Miki-cho, Kita-gun, Kagawa 761-0793, Japan

## Abstract

*Introduction.* Osteoma is a benign, slowly growing, asymptomatic, osteogenic neoplasm. Osteoma of a bone other than the skull and facial bones is extremely rare. An extremely rare case of parosteal osteoma is reported. *Case Presentation.* A 51-year-old woman presented with a large mass in the left supraclavicular fossa. Radiographs and computed tomography revealed a well-defined, 9 × 6 cm, lobed mass in the midportion of the clavicle. Magnetic resonance imaging revealed that it had the same density as cortical bone. An open biopsy was performed to rule out malignant bone tumours, and parosteal osteoma was diagnosed. Four years after the biopsy, the patient was asymptomatic. *Conclusion.* A rare case of parosteal osteoma of the clavicle was described. Open biopsy is required to rule out a malignant bone tumour, even if parosteal osteoma is suspected based on the clinical course and imaging findings.

## 1. Introduction

Osteomas arise from cancellous or compact bone and are often referred to as benign osteogenic lesions. Clinically, they appear circumscribed, usually rounded and protuberant, and are characterized by very slow, continuous growth [1]. Osteomas are bone lesions with different onset types that may be categorized as those involving the skull and facial bones, exostoses or bone islands, or long bone superficial osteomas (parosteal osteoma or juxtacortical osteoma) [2, 3]. Osteomas are most commonly located in the skull and facial bones. Primary extracranial osteomas are extremely rare, with a prevalence of 0.03% of biopsied primary bone tumours [3]. An osteoma is usually a solitary lesion, but there may be multiple lesions in patients with Gardner syndrome. These lesions are associated with intestinal polyps, fibromatous and other lesions of connective tissue, and epidermal cysts [3, 4]. Histologically, osteomas are composed of dense sclerotic lamellar bone similar to that in cortical bone. A rare case of parosteal osteoma of the clavicle is reported.

## 2. Case Presentation

A 51-year-old woman presented with a large mass in the left supraclavicular fossa. The mass was incidentally found on a chest radiograph approximately 30 years earlier. The patient had been asymptomatic but reported that the mass had gradually increased in size.

Physical examination revealed an ill-defined, 9 × 6 cm, rough-surfaced, bony-hard mass in the anterosuperior aspect of the left clavicle. Shoulder movement was not limited, and no stigmata of Gardner's syndrome were noted.

**Figure 1:**
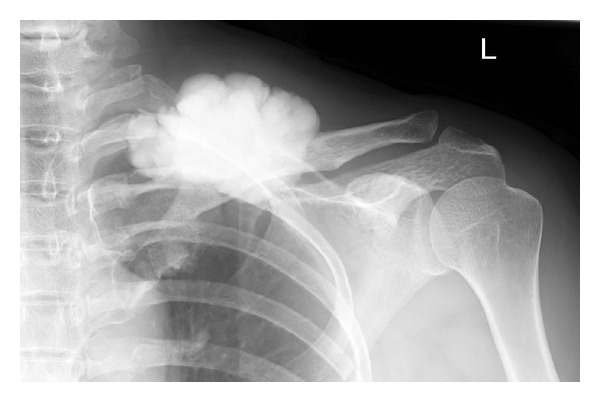
Radiographs reveal a well-defined, lobed mass in the midportion of the clavicle.

**Figure 2:**
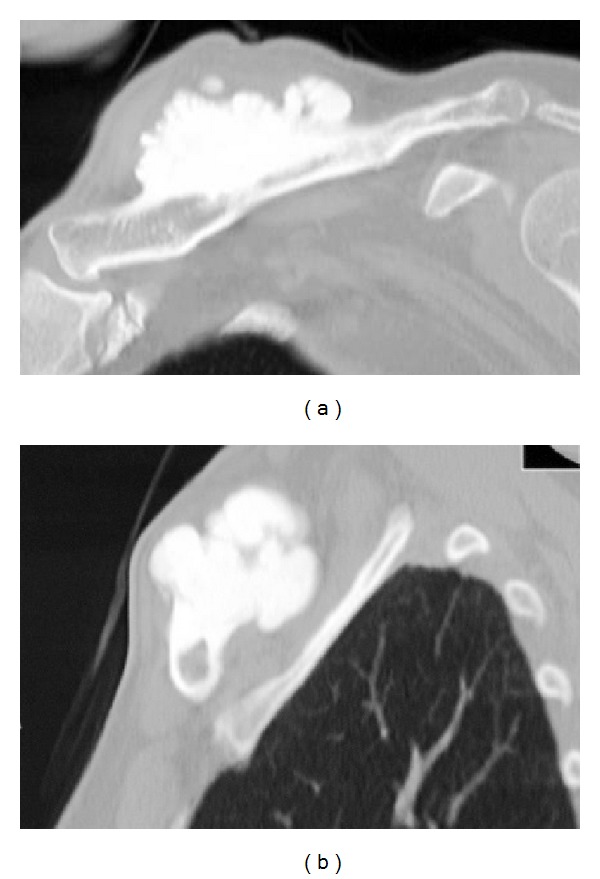
Computed tomography scan reveals that the heavily ossified mass is attached to the cortex and has no areas of lucency. The cortex underlying the osteoma is intact. (a) Coronal image of the left clavicle; (b) sagittal image of the left clavicle.

**Figure 3:**
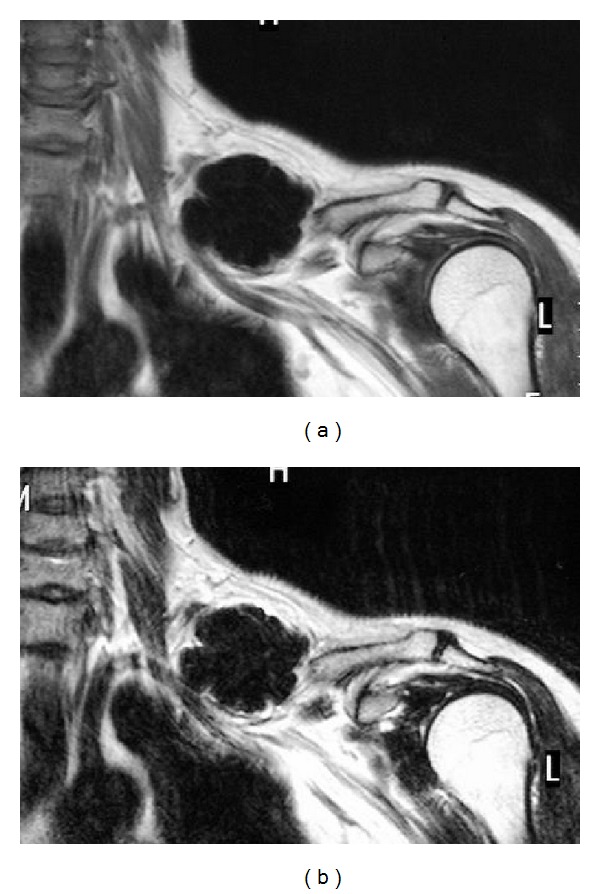
Magnetic resonance imaging reveals that the lesion has the same density as cortical bone. (a) A coronal T1-weighted image shows that the lesion has low signal intensity located in the midportion of the clavicle. (b) This coronal T2-weighted image shows the lesion with low signal intensity.

Radiographs revealed a well-defined, 9 × 6 cm, lobed mass in the midportion of the clavicle bone (Figure 1). A computed tomography (CT) scan revealed that the heavily ossified mass was attached to the cortex, with no areas of lucency. The cortex underlying the osteoma was intact (Figures 2(a) and 2(b)). Magnetic resonance imaging (MRI) showed that it had the same density as cortical bone (Figures 3(a) and 3(b)). Bone scintigraphy showed an uptake area in the clavicle at the same place as the lobed mass.

**Figure 4:**
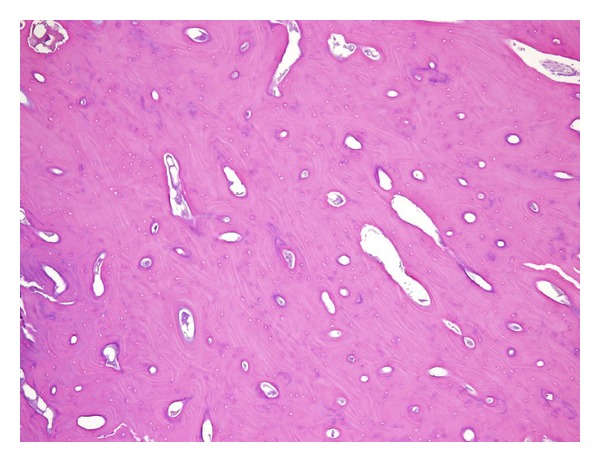
Histological examination shows that the tissue consists of dense sclerotic lamellar bone.

An open biopsy was performed to rule out parosteal osteosarcoma. Histologically, the resected tissue was composed of dense sclerotic lamellar bone, and no evidence of malignancy was found. Thus, a diagnosis of parosteal osteoma was made (Figure 4).

Four years after the biopsy, the patient remained asymptomatic, even though the tumour appeared to be growing slightly.

## 3. Discussion

Osteoma is a benign osteogenic lesion with very slow growth that is classified into the following three types based on location or clinicopathologic features: (1) skull and facial bones; (2) exostoses or bone islands; and (3) long bone superficial osteomas (parosteal osteoma or juxtacortical osteoma) [2, 5].

A parosteal osteoma of the long bone may be an asymptomatic incidental finding, or the patient may have noted a mass lesion that enlarged gradually. It is thought that it originates as ossification in intramembranous bone and can be seen at almost any age, with a focus on the 4th and 5th decades.

Radiographs reveal homogeneous, well-defined, smooth round, or lobulated margins. Typically, a CT scan shows that the heavily ossified mass is attached to the cortex, with no areas of lucency.

Primary extracranial osteomas are extremely rare, representing only 0.03% of biopsied primary bone tumours [6]. To the best of our knowledge, only three reports of osteomas involving the clavicle have been reported, one case reported by Meltzer et al. [4], two cases reported by Schajowicz [7] and one case reported by Yamamoto et al. [8].

The differential diagnosis of parosteal osteoma should include tumoural calcinosis, osteochondroma, myositis ossificans, parosteal osteosarcoma and parosteal chondrosarcoma. It is especially important to rule out a malignant bone tumour. Unlike conventional osteosarcoma, this type of tumour most commonly occurs in patients in the 3–5th decades of life. Although low-grade, the lesion can infiltrate the underlying cortex and extend into the medullary cavity; it also has a propensity to recur [9]. The appearance of scintigraphy can help to differentiate osteosarcoma. Yamamoto et al. [8] reported SPECT/CT imaging in bone scintigraphy and described osteoma usually presents as a homogeneous and dense lesion without an accompanying soft-tissue mass, cortical destruction, or intramedullary invasion. In our case, the imaging of bone scintigraphy and CT showed homogeneous mass without soft-tissue mass, cortical destraction and intramedullary invasion.

The diagnosis may be difficult and risky with a small biopsy sample; however, in conventional osteoma, surgical intervention is usually only warranted in symptomatic patients. On the other hand, it is not unusual for parosteal osteosarcoma to be treated with definitive operative resection without a previous biopsy [8].

Osteoma is a benign disease. If areas of lucency are not seen on radiography, and histological examination does not reveal spindle cell proliferation, a diagnosis of osteoma can be considered. Once the diagnosis of parosteal osteoma has been made, wide resection is no longer necessary because recurrence is rare [10] and malignant transformation has never been reported [11]. Therefore, only careful follow-up or marginal resection may be sufficient for this lesion.

## 4. Conclusion

A rare case of parosteal osteoma of the clavicle was described. Open biopsy is required to rule out a malignant bone tumour, even if parosteal osteoma is suspected based on the clinical course and imaging findings. The patient was followed up for four years and remained asymptomatic throughout. The diameter of the tumour increased slightly over the 4-year followup. Since the growth potential of osteoma remains unclear, the patient requires close clinical and radiographic followup.
